# Preventive effect of cranberries with high dose of proanthocyanidins on urinary tract infections: a meta-analysis and systematic review

**DOI:** 10.3389/fnut.2024.1422121

**Published:** 2024-11-28

**Authors:** Zheyu Xiong, Yongli Gao, Chi Yuan, Zhongyu Jian, Xin Wei

**Affiliations:** ^1^Department of Urology and Institute of Urology (Laboratory of Reconstructive Urology), West China Hospital, Sichuan University, Chengdu, China; ^2^Department of Emergency Medicine, West China Hospital of Sichuan University, Chengdu, China; ^3^Department of Emergency Medicine, West China School of Nursing, Sichuan University, Chengdu, China; ^4^Department of Pediatric Surgery, West China Hospital, Sichuan University, Chengdu, China

**Keywords:** meta-analysis and systematic review, cranberry, proanthocyanidins, urinary tract infections, dose

## Abstract

**Introduction:**

One of the most prevalent bacterial diseases in both the general population and hospitals are urinary tract infections (UTIs). There is still conflicting scientific data about the usefulness of cranberry products in preventing UTIs. Our meta-analysis investigated whether the content of the main component, proanthocyanidins (PACs), in cranberries affects their ability to prevent UTIs.

**Methods:**

The average daily intake of PACs has already been reported in previous randomized controlled trials (RCTs) that intended to investigate the effectiveness of cranberry in preventing UTIs, which were collected in our meta-analysis. The results were reported as the number of participants with UTIs. Random effect or fixed effect models were chosen for statistical analysis based on the heterogeneity.

**Results:**

Ten RCTs that matched the requirements were included. The results showed that when the daily intake of PACs was at least 36mg, the risk of UTIs was reduced by 18% (RR = 0.82, 95% CI = 0.69–0.98, *p* = 0.03). But when the daily intake of PACs was less than 36 mg, there was no statistical significance risk decrease (*p* = 0.39). The results of the sub-group analysis showed that cranberries only significantly reduced the risk of UTIs when the duration of cranberry product use falls between 12 and 24 weeks (RR = 0.75, 95% CI = 0.61–0.91, *p* = 0.004). Additionally, cranberries also significantly reduced the risk of UTIs only in subgroups that just included females (RR = 0.84, 95% CI = 0.71–0.98, *p* = 0.02).

**Discussion:**

These findings showed a strong correlation between the daily use of the active ingredient PACs found in cranberry products and the prevention of UTIs. Our meta-analysis is the first to show that there are minimum daily PAC consumption intake levels in cranberry products and length of use considerations that are needed to achieve clinically relevant UTI prevention benefits.

**Systematic review registration:**

PROSPERO (CRD42023385398).

## 1 Introduction

One for the leading reasons of emergency urological visits is UTIs (1). A UTI will affect one in three women over the age of 18, and many of them also have recurrent UTIs, which will can cause serious distress in patients’ lives (2). Antibiotics are used to treat UTI but can also be prescribed at low doses over extended periods to prevent infection; however, the emergence of antibiotic resistance has prompted clinicians and patients to seek non-antibiotic alternatives for reducing the risk of recurrent UTI (1). Therefore, it is urgent to seek a safe and reliable substance to prevent the occurrence of UTIs. Numerous RCTs have evaluated cranberry intake and the effects on UTI prevention with a meta-analysis in 2012 finding inconsistent clinical results among studies (3). The inconsistencies were mostly attributed to the use of non-standardized cranberry preparations, poor statistical power, and a lack of compliance. Some recent studies have shown the importance of using cranberry products that contain proanthocyanidins (PACs) from the juice, not the pulp (4), and that these PACs at intake levels of 36 mg can result in the production of urine that has anti-adhesive properties that keep UTI-causing bacteria from attaching in the bladder where they can cause infections (4). But these *ex vivo* findings regarding the 36-mg intake dose must be validated through human intervention trials for infection-prevention benefits. A recent 2023 meta-analysis and systematic review of 50 clinicals found that cranberry consumption prevented and reduced risk of recurrent UTI in women, children and certain at-risk people (5). This meta-analysis was able to confirm the ability of daily cranberry intake for UTI prevention in these groups, but there were different PAC intakes in each study making it difficult to identify the optimal daily PAC level needed to prevent UTI recurrence. In addition, there is no consensus on the length of time cranberry should be consumed for the benefits or if there is a difference in response to cranberry intake between men and women.

Given the resistance associated with overuse of antibiotics to treat UTIs and the effectiveness that cranberry has to prevent the infections in a natural way, more clarification is needed regarding PAC dose, length of intervention and differences in response to cranberry by gender. We thus performed this meta-analysis of RCTs that reported PACs dosages to determine the influence of PAC dose, intervention length and gender on achieving clinical efficacy in preventing recurrence of UTIs.

## 2 Methods

Our protocol has been registered in PROSPERO (CRD42023385398).

### 2.1 Search strategy

Using a predetermined search strategy, we conducted thorough searches in PubMed, Embase, and the Cochrane Library of Systematic Reviews up until November 2022. Key words of search strategy include “urinary tract infection (UTI),” “cranberry,” “proanthocyanidin (PAC).” The Boolean operators “OR” and “AND” were used to combine all words. Specific search strategies are shown in Supplementary Table S1.

### 2.2 Eligibility criteria and study selection

We used the PRISMA flowchart to record the study selection process (6). Before reading the whole papers, titles and abstracts for full-text publications were checked for eligibility. The publications were all reviewed by two writers using the following inclusion and exclusion criteria.

The following criteria were satisfied by original articles to be included: (1) we only included RCTs as part of the study design; (2) RCTs analyzed the relationship between cranberry and any type of UTIs from different participants; (3) only studies comparing cranberry-containing products to a placebo (beverage) or non-placebo control group for the applied intervention were considered; (4) trials reported the PACs content in cranberry products; (5) the outcome indicators of trials included the number of participants experiencing UTIs.

The original articles that were chosen for inclusion fit the following criteria: (1) trials analyzed the relationship between cranberry in combination with other substances and UTIs; (2) animal studies, case studies, systematic reviews, and trials with incomplete evidence were all disregarded.

### 2.3 Data extraction and methodological assessment

One author extracted the data, and another author verified it. Consensus was used to resolve disputes. We gathered the initial author’s name, the year the work was published, the length of the treatment, the populations, the intervention of the experimental and control groups, the dosage of the PACs, and the conclusion from each eligible RCT. And we also extracted the number of participants experiencing UTIs or recovering from UTIs at the endpoint of trials which was used for statistical analysis.

Quality assessment of the included trials was done using the Cochrane risk of bias method, which has 7 domains (7).

### 2.4 Outcome definitions

Outcome definition: number of UTIs patients in the treatment and control groups at the end of the study. When expressed as a percentage in the original study, convert this value to the corresponding number of patients.

### 2.5 Data analysis

Using Review Manager 5.4.1 software and Cochrane guidelines, a meta-analysis of RCT results was carried out. We used odds ratios (OR) and their associated 95% confidence intervals (CI) to assess outcomes, and considered a *p*-value less than 0.05 to be statistically significant. Heterogeneity was evaluated using the *I*^2^ test. If significant heterogeneity was not present (*I*^2^ < 50%), we used fixed effects models to pool outcomes. Conversely, when significant heterogeneity was present (I2 ≥ 50%), we used random effects models for analysis (8, 9). By using predetermined variables, we performed subgroup analyses. In addition, to address potential multiple comparison issues in subgroup analyses, we also used Bonferroni correction to control the family-wise error rate.

## 3 Results

### 3.1 General characteristics of included RCTs

**Figure 1:**
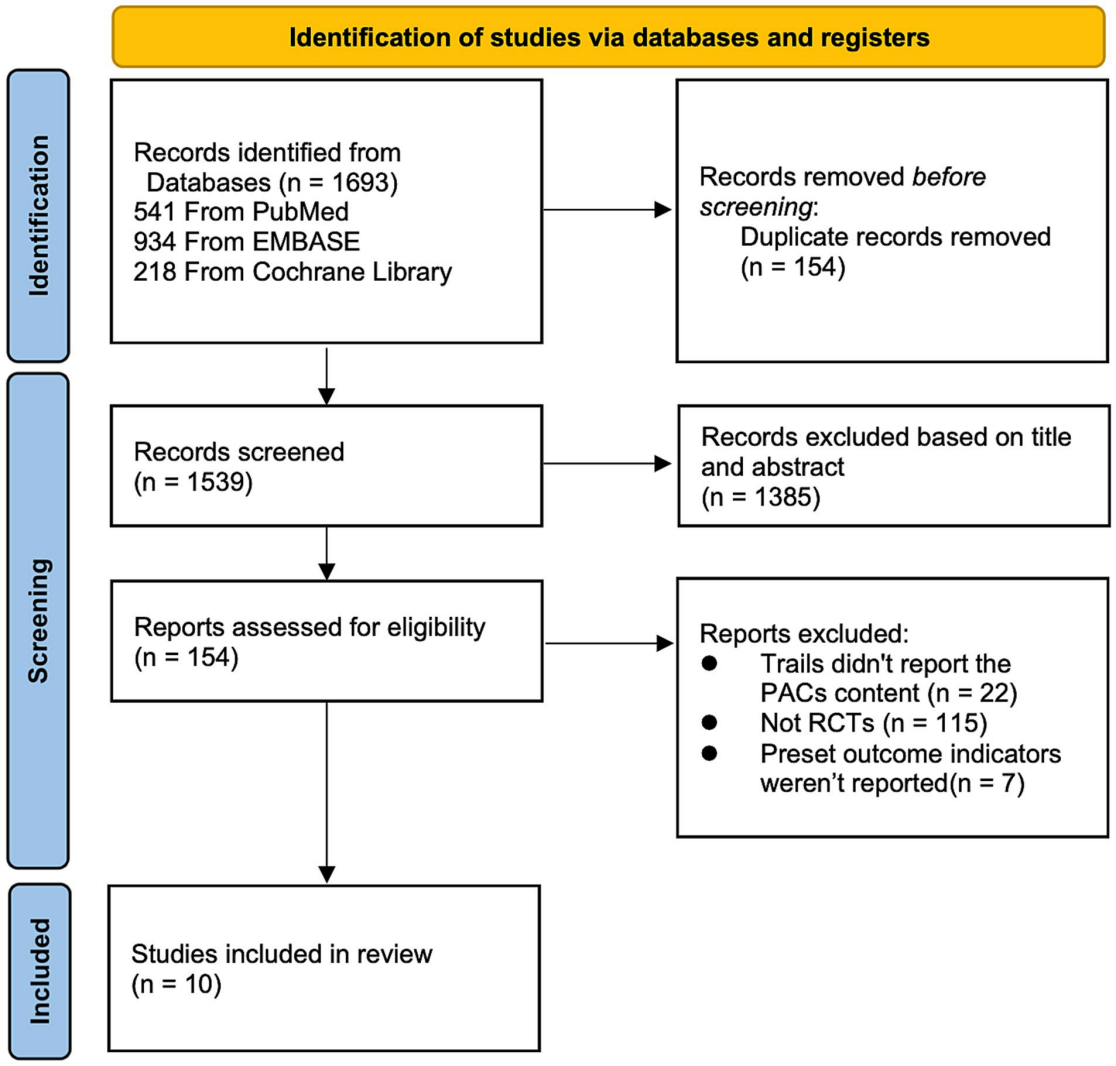
Flowchart of the study selection for the systematic review and meta-analysis.

From all the databases, the initial literature search turned up 1,693 articles (541 from PubMed, 934 from Embase, and 218 from Cochrane Library). There were 1,539 records left after the duplicates were eliminated. After eligibility checks, 10 pertinent studies were chosen for the meta-analysis (10–19). All included RCTs determined the sample size for each group through power analysis to ensure the statistical power of their own studies. The flow chart of research selection for systematic evaluation and meta-analysis is shown in Figure 1. The features of the selected RCTs are outlined in Table 1.

**Table 1:** Characteristics of selected RCTs.

Author	Year	Treatment duration	Participants	Experimental group	Proanthocyanidin dose	Control group	Primary outcome	Secondary outcome	Conclusion
PACs <36 mg/day									
Temiz et al. (10)	2018	12 weeks	40 patients underwent ileal conduit diversion aged 18 + years	Cranberry capsule (containing 400 mg of the product, with 9 mg PACs) twice daily (*n* = 20)	18 mg/day	Placebo (*n* = 20)	Number of new symptomatic urinary tract infections	White blood cell count + CRP values	Cranberry capsules is effective in the prevention of UTIs
Gunnarsson et al. (11)	2017	2 weeks	80 women aged 60 + with hip fracture	Two cranberry powder capsules (550 mg containing 4.19 mg PACs each) three times a day (*n* = 52)	12.57 mg/day	Placebo (*n* = 40)	Number of new symptomatic urinary tract infections	NA	This study could not demonstrate a protective effect of cranberry concentrate on hospital-acquired UTIs in elderly women with hip fracture and urinary catheterization
Caljouw et al. (12) (low UTI risk)	2014	48 weeks	412 long-term care facilities residents	Cranberry capsule (containing 500 mg of the product, with 9 mg PACs) twice daily (*n* = 205)	18 mg/day	Placebo (*n* = 207)	Number of new symptomatic urinary tract infections	Person-Days at Risk + Incidence Per 100 Person Years at Risk	No difference in incidence of UTI was found in residents with low UTI risk
Caljouw et al. (12) (high UTI risk)	2014	48 weeks	516 long-term care facilities residents with long-term catheterization (>1 month), diabetes mellitus, or at least one UTI in the preceding year	Cranberry capsule (containing 500 mg of the product, with 10 mg PACs) twice daily (*n* = 253)	19 mg/day	Placebo (*n* = 263)	Number of new symptomatic urinary tract infections	Person-days at risk + incidence per 101 person years at risk	Participants with high UTI risk taking cranberry capsules twice daily reduces the incidence of clinically defined UTI
Mcmurdo et al. (13)	2005	48 weeks	376 older hospital patients aged 60 + years	150 mL cranberry juice twice daily (containing 0.838 mg of PAC, *n* = 187)	0.838 mg/day	Placebo beverage (*n* = 189)	Number of new symptomatic urinary tract infections	Infections with *Escherichia coli*	Cranberry juice ingestion is not effective in the prevention of UTI in elderly hospital patients
PACs ≥36 mg/day									
Babar et al. (14)	2021	24 weeks	145 healthy, adult women with a history of recurrent UTIs	Cranberry proanthocyanidins (2 × 18.5 mg daily, *n* = 72)	37 mg/day	Cranberry proanthocyanidins (2 × 1 mg daily, *n* = 73)	Number of new symptomatic urinary tract infections	Symptomatic urinary tract infection with pyuria or bacteriuria	High dose of proanthocyanidins may have a preventive impact on symptomatic urinary tract infection recurrence in women who experienced less than 5 infections per year
Mooren et al. (15)	2020	6 weeks	210 women aged 18 + undergoing pelvic organ prolapse or incontinence surgery	Two cranberry capsules (containing 36 mg of PACs each) daily (*n* = 105)	72 mg/day	Placebo (*n* = 105)	Number of new symptomatic urinary tract infections	NA	This trial shows no beneficial effect of adequately dosed cranberry prophylaxis in women undergoing pelvic floor surgery
Ledda et al. (16)	2017	8 weeks	36 healthy, juvenile age subjects (between 12 and 18 years of age) with a history of recurrent UTIs	Standard management (SM, lifestyle and hygiene advice) + Cranberry proanthocyanidins (36 mg daily, *n* = 19)	36 mg/day	SM (*n* = 17)	Number of new symptomatic urinary tract infections	NA	Highly standardized cranberry extract (Anthocran^®^) can be as prophylaxis in young healthy subjects suffering by recurrent UTIs
Letouzey et al. (17)	2017	10 days	255 women undergoing pelvic surgery aged 18 + years	One cranberry capsule (containing 36 mg of PACs) daily (*n* = 132)	36 mg/day	Placebo (*n* = 123)	Number of new symptomatic urinary tract infections	Bacteriuria within 40 days after surgery + Rates of *E. coli* infections by day 15 and day 40	Cranberry capsules do not reduce the risk of bacteriuria after pelvic surgery
Juthani-mehta et al. (18)	2016	48 weeks	185 nursing home women resident aged 65 + years	Two cranberry capsules (containing 36 mg of PACs each) daily (*n* = 92)	72 mg/day	Placebo (*n* = 93)	Number of new symptomatic urinary tract infections	Number of symptomatic UTI, all-cause death, all-cause hospitalization	Administration of cranberry capsules compared with placebo resulted in no significant difference in presence of bacteriuria plus pyuria over 1 year
Takahashi et al. (19)	2013	24 weeks	213, 20 to 79 years old women with acute exacerbation of acute uncomplicated cystitis or chronic complicated cystitis with a history of multiple relapses of UTIs	Cranberry juice (125 mL with proanthocyanidin >40 mg daily, *n* = 107)	>40 mg/day	Placebo beverage (*n* = 106)	Number of new symptomatic urinary tract infections	NA	There was no significant difference in the relapse rates of UTI between groups A and P. Cranberry juice prevented the recurrence of UTI in a limited female population (aged 50 years or more) with 24-week intake of the beverage

Among the 10 RCTs included, 3 studies took the occurrence of acute urinary tract symptoms as an indicator to measure the recurrence of UTIs (14, 16, 19), 3 studies took positive urine culture results as the indicator (10, 13, 17), and the remaining 4 studies took both acute urinary tract symptoms and urine culture results into consideration (11, 12, 15, 18). Different RCTS include different populations at risk of UTIs, including women with recurrent UTIs, women susceptible to UTIs after various surgeries, nursing home women residents, long-term care facilities residents, and elderly hospitalized patients. The follow-up time of each study also varied, with the longest reaching 48 weeks and the shortest only 10 days. Although participants consumed cranberries from different sources (juice, capsules, etc.), actual daily PACs were recorded for each cranberry product, with six groups having daily PACs of 36 mg or more (14–19) and the remaining five groups having daily PACs of less than 36 mg (10–13). Eight of the studies used either a formulated placebo or a placebo drink, and of the remaining two studies, one used a low dose of PACs (2 mg/ day) as a control, another used standard management (including associated lifestyle and hygiene advice) as a control. Of the 10 studies, six were conducted in Europe (the Netherlands, Sweden, Italy, France and the United Kingdom) (11–13, 15–17), two in North America (Canada and the United States) (14, 18), and the remaining two in Asia (Turkey and Japan) (10, 19).

### 3.2 Meta analysis

**Figure 2:**
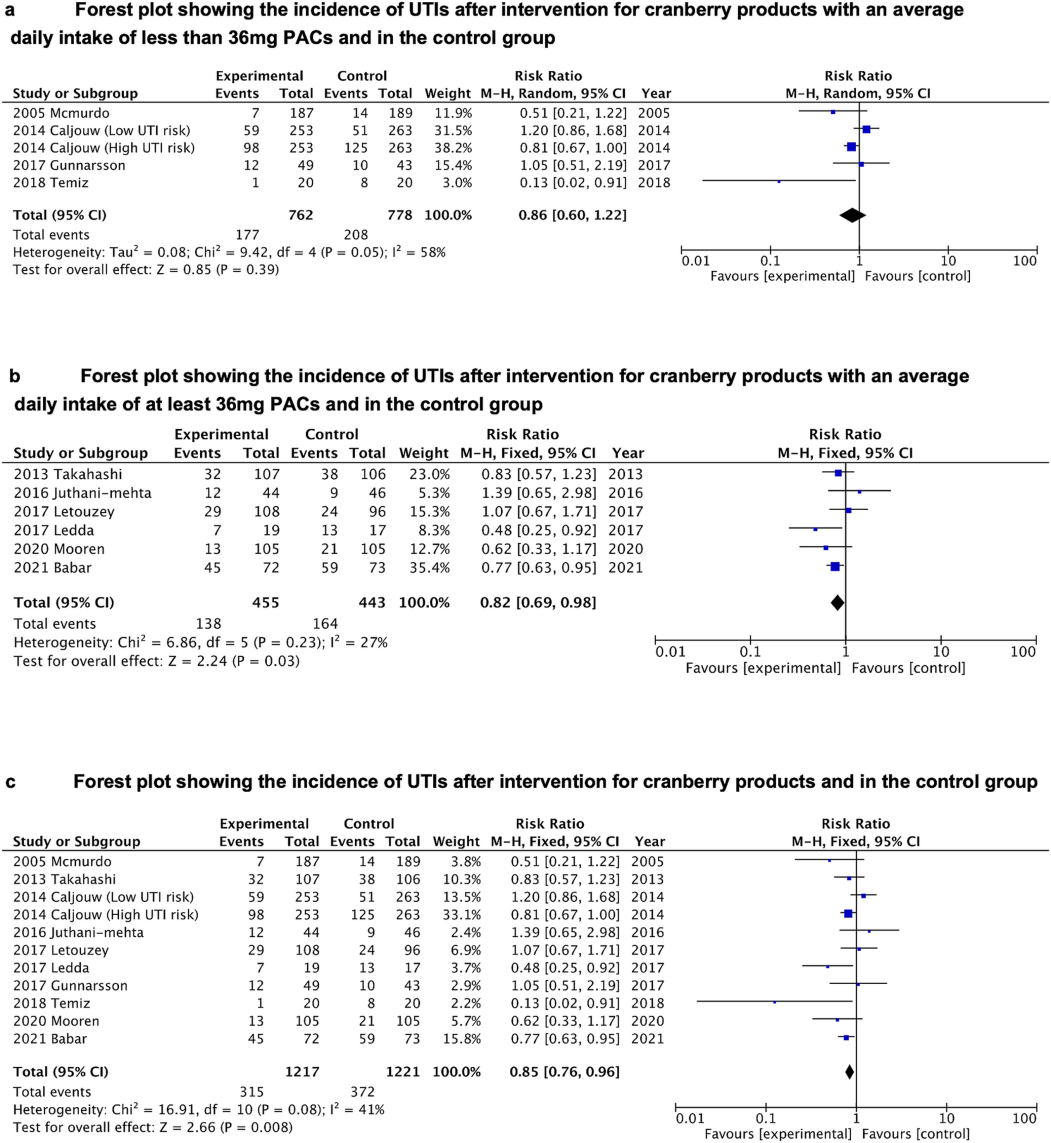
**(A)** Forest plot showing the incidence of UTIs after intervention for cranberry products with an average daily intake of less than 36 mg PACs and in the control group. **(B)** Forest plot showing the incidence of UTIs after intervention for cranberry products with an average daily intake of at least 36 mg PACs and in the control group. **(C)** Forest plot showing the incidence of UTIs after intervention for cranberry products and in the control group.

In order to determine whether the amount of PACs in various cranberry products affected how well they prevented UTIs, we performed a meta-analysis of the original trials that used PACs at dosages lower than 36 mg per day and more than 36 mg per day. The number of recurrent UTIs was used as an outcome indicator in a meta-analysis of 10 RCTs, with the findings displayed in Table 1. PACs intakes were less than 36 mg/day in 5 of the original studies and at least 36 mg/day in 6 of the original studies. There were 2,438 individuals in the analysis (1,217 from the PACs group and 1,221 from the control group). In the grouping with daily PACs intake less than 36 mg, there was no statistically noteworthy distinction between the experimental group compared to the control (*p* = 0.39). The results showed moderate heterogeneity (*I*^2^ = 58%) (see Figure 2A). In comparison to controls, cranberry products decreased the risk of UTI by 18% among those who consumed 36 mg or more of PACs per day. This difference was statistically significant (*p* = 0.03) (see Figure 2B).

We also conducted an overall meta-analysis of 10 RCTs. In the entire study, it was discovered that cranberry products significantly (*p* = 0.008) decreased the incidence of UTI by 15% when compared with controls (RR = 0.85, 95% CI = 0.76–0.96) (see Figure 2C).

### 3.3 Sub-group analysis

**Figure 3:**
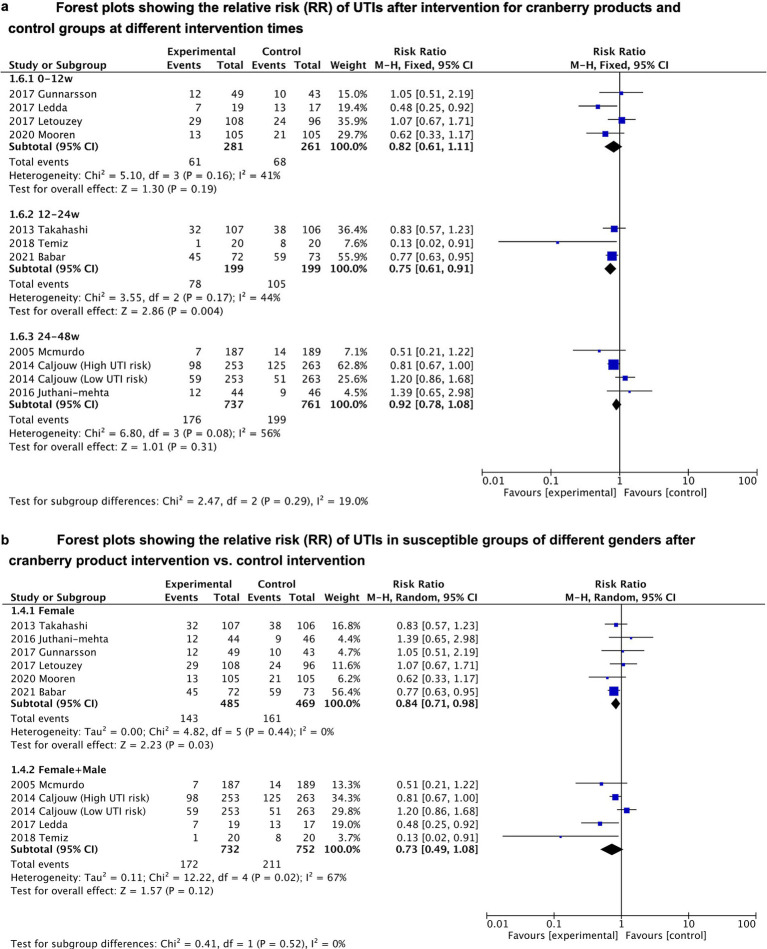
**(A)** Forest plots showing the relative risk (RR) of UTIs after intervention for cranberry products and control groups at different intervention times. **(B)** Forest plots showing the relative risk (RR) of UTIs in susceptible groups of different genders after cranberry product intervention vs. control intervention.

Subgroup analyses were conducted to assess the effect of duration of cranberry product use and gender of the included population on cranberry effectiveness in preventing UTIs (see Figures 3A,B). The results of the subgroup analysis showed that cranberries only significantly reduced the risk of UTIs when the duration of cranberry product use falls between 12 and 24 weeks (RR = 0.75, 95%CI = 0.61–0.91). After applying the Bonferroni-corrected *p*-value, the conclusion still demonstrates statistically significant differences (*p* = 0.004 < 0.05/3). There was no significant association between cranberries and UTI risk when the duration of use was too short (less than 12 weeks) or too long (24 to 48 weeks). Additionally, cranberries also significantly reduced the risk of UTIs only in subgroups that just included females (RR = 0.84, 95% CI = 0.71–0.98), rather than in subgroups that included both males and females (*p* = 0.12). However, the conclusion derived from gender-related sub-group analysis was questioned when the Bonferroni-corrected *p*-value was introduced (*p* = 0.03 > 0.05/2).

### 3.4 Quality assessment

**Figure 4:**
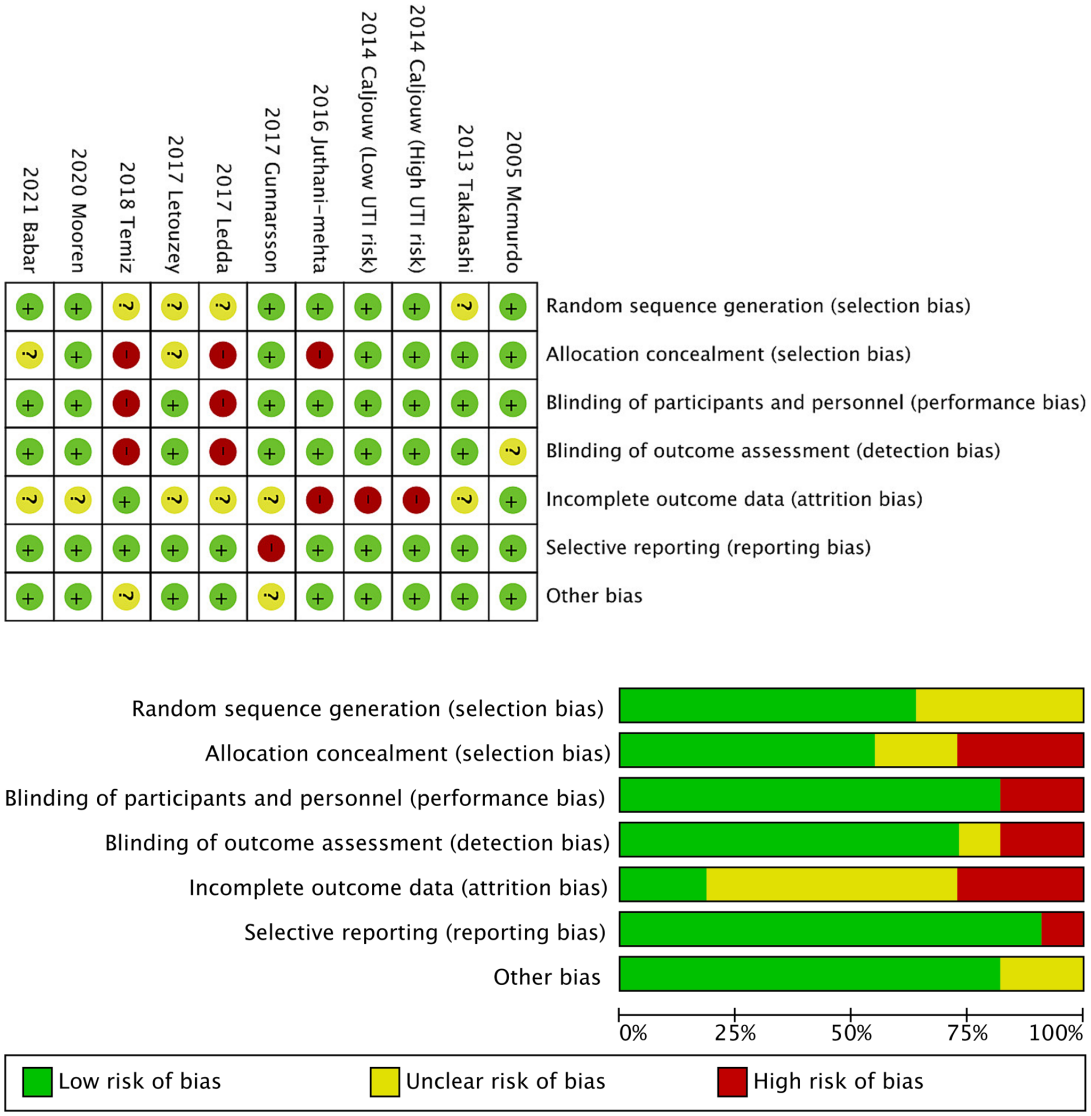
The risk assessment between studies.

The risk of bias assessment results for the included 10 RCTs, along with the bias risk graph presented in percentage form, are all displayed in Figure 4. Six studies thoroughly described their randomization methods, while the remaining four studies were identified as unclear risk due to limited details provided in their research designs and methods. Only five studies reported sufficient allocation concealment, with three research works identified as high risk. Performance bias was predominantly low risk (80%), with these eight studies detailing the blinding process of subjects or researchers, while the remaining two studies were identified as high risk. Detection bias was also predominantly low risk (70%), with two study identified as high risk. The risk of attrition bias was unclear in six studies (60%), and only two research was regarded as at low risk. Only one study was identified as having a high risk of reporting bias, while the majority of studies (90%) provided data on their study results and registered their trials in publicly available trial registries. Only two studies were rated as having an unclear risk of other biases, while the remaining eight studies showed no significant signs of other biases.

## 4 Discussion

We acknowledge that this is the first meta-analysis to assess the impact of PACs dose on the recurrence of UTIs. While the overall results of the analysis indicate that cranberry products reduce the risk of UTIs, a more detailed analysis reveals that the risk of UTI recurrence significantly decreases only when the daily intake of the active component PACs in cranberries is at least 36 mg. Furthermore, subgroup analysis also indicates that the risk of UTIs is significantly reduced only when consuming cranberry products continuously for 12 to 24 weeks. Therefore, our works suggest that the protective effect of cranberries against recurrent UTIs may be associated with the daily intake of PACs, and the key to the protective efficacy of cranberries may also lie in the appropriate duration of product usage.

While a substantial amount of epidemiological studies, intervention trials, and meta-analyses have confirmed the effectiveness of cranberry products in preventing UTIs, controversy still exists (20–23). The differing compositions and dosages of cranberry products used in various intervention studies may be a significant factor contributing to the discrepancies observed among studies (24). Some previous meta-analyses have also found that cranberry capsules made from cranberry extract appear to be more effective in reducing the risk of UTIs in subgroup analyses. They suggest that cranberry extract capsules may contain higher levels of active ingredients (22, 23). Moreover, results from previous *in vitro* studies also suggested that the active component PACs in cranberries can effectively inhibit the adhesion of pathogenic *Escherichia coli* to uroepithelial cells and that a daily intake of at least 36 mg of PACs can produce a significant anti-adhesion effect (25). Based on this, our study categorized RCTs reporting daily PACs intake into two groups based on different intake levels. And the results indicated that cranberries significantly reduce the risk of UTIs only when the daily intake of PACs is not less than 36 mg.

Although PACs have been considered the primary reason for cranberries’ UTI prevention properties in recent decades, the specific mechanisms by which they operate are still being researched (26). While PACs can prevent the adhesion of pathogenic *E. coli* to uroepithelial cells under *in vitro* conditions, this finding has been challenged by recent research indicating limited absorption of PACs ingested by humans due to metabolism by gut microbiota and low levels of PACs in urine (27, 28). Further research has shown that a significant amount of phenolic metabolites produced by microbial breakdown of PACs in the gut can be absorbed by the body and excreted through urine (29). These phenolic metabolites may inhibit bacterial adhesion to uroepithelial cells at the initial stages, thereby playing a role in preventing bacterial colonization and the progression of UTIs (28). Based on our research findings, it can be inferred that high doses of PACs intake may enhance anti-adhesion functions in the urinary tract by increasing the production and absorption of phenolic metabolites, thereby reducing the risk of UTIs. It is important to note that there are different analytical methods used to determine PAC levels in cranberry products and some methods are not accurate and falsely overstate the amount of PACs by including detected contaminants. The accepted method used to target 36 mg PAC in products in the cranberry industry is the DMAC method using the procyanidin A2 standard (30, 31). Going forward, it would be important for clinical trials using cranberry products to specifically state the method and reference standard used to quantify the PAC levels so accurate efficacy assessments by dose can be made.

The current recommendation by the European Association of Urology suggests the continuous use of cranberry products as an alternative method for preventing UTIs. Taking this into consideration, we conducted a subgroup analysis on the duration of cranberry product use. The relevant results indicate that the risk of UTIs is significantly reduced only when cranberry products are consumed continuously for 12 to 24 weeks. However, given that the longest duration of cranberry use in current studies is only 1 year, our research conclusions can only suggest that another key aspect of cranberry’s protective effect may lie in the appropriate duration of cranberry product use. This does not negate the potential benefits that long-term cranberry product use may offer. Therefore, future RCTs may need to encompass significantly longer periods. When the duration of cranberry product usage is less than 12 weeks, the reduction in UTI risk is also not significant. This may be attributed to the possibility that after short-term use of cranberry products, PACs and their metabolites have not fully exerted anti-adhesive activity before being excreted from the body.

In summary, our study confirms that the protective effect of cranberries against UTIs may be closely related to the daily intake of PACs. Additionally, another key factor in cranberries exerting its protective effect against UTIs might be the appropriate duration of cranberries product use. However, there are also some limitations to the study. Firstly, the number and sample sizes of RCTs that currently meet the inclusion criteria are limited, and the research quality varies. These factors can make our research conclusions easily change as the number of included studies and sample sizes increase, along with the inclusion of high-quality studies. Therefore, future research will still require large sample sizes and high-quality RCTs to further investigate how different doses of PACs affect the effectiveness of cranberry products in preventing UTIs. Secondly, the inevitable issue of excessive follow-up loss in the original studies may result in a lower true sample size than the one initially calculated through power analysis, thereby affecting statistical power. Thirdly, the original studies included had different criteria for the occurrence of UTIs, and RCTs that only included symptoms when determining the prevalence of UTIs were likely to overestimate the prevalence of UTIs in vulnerable groups. This could potentially impact the assessment of the true effectiveness of cranberries in preventing UTIs. Lastly, since current studies have only assessed cranberry use for up to 1 year, the results of sub-group analyses may not be sufficient to rule out the potential benefits of long-term cranberry product use. Future RCTs may need to cover longer durations of cranberry interventions.

## 5 Conclusion

Our meta-analysis results for the first time validated a strong correlation between the daily intake of the active ingredient PACs in cranberry products and the prevention of UTIs. The results of subgroup analysis also suggested that another key aspect of cranberries’ protective effect on UTIs may lie in the appropriate duration of cranberries product use. This may indicate that clinicians need to consider both the intake dosage of PACs and the duration of use when advising patients on using cranberry products for preventing UTIs. In the future, large sample sizes and high-quality RCTs will still be needed to further investigate how different doses of PACs and duration of use affect the effectiveness of cranberry products in preventing UTIs.

## Data Availability

The original contributions presented in the study are included in the article/Supplementary material, further inquiries can be directed to the corresponding author.
