# Supplementary material for: Preventive effect of cranberries with high dose of proanthocyanidins on urinary tract infections: a meta-analysis and systematic review
Source: Front Nutr. 2024 Nov 28;11:1422121. doi: 10.3389/fnut.2024.1422121 (PMC11635990; doi:10.3389/fnut.2024.1422121)
Supplement: Supplementary file 1 [file Table_1.DOCX]

***Supplementary Material***

**Preventive Effect of Cranberries with High Dose of Proanthocyanidins on Urinary Tract Infections: A Meta-analysis and Systematic Review**

Zheyu Xiong^1#^, Yongli Gao^2#^, Chi Yuan^3^, Zhongyu Jian^1^, Xin Wei^1*^

*** Correspondence:** Xin Wei; Mailing address: Department of Urology and Institute of Urology (Laboratory of Reconstructive Urology), West China Hospital, Sichuan University, No.37 Guoxue Alley, Wuhou District, Chengdu City, Sichuan Province, PR China. E-mail address: weixinscu@scu.edu.cn.

**Supplementary Table 1: Search strategy**

**Medline (through PubMed)**

| Query # | Query | Result |
| --- | --- | --- |
| 1. | Vaccinium macrocarpon[Mesh] OR Vaccinium macrocarpons[tiab] OR macrocarpon, Vaccinium[tiab] OR Cranberry[tiab] OR Cranberries[tiab] | 2146 |
| 2. | Urinary Tract Infections[Mesh] OR Infection, Urinary Tract[tiab] OR Infections, Urinary Tract[tiab] OR Tract Infection, Urinary[tiab] OR Tract Infections, Urinary[tiab] OR Urinary Tract Infection[tiab] | 78,501 |
| 3. | 1 AND 2 | 541 |

**Embase (Ovid platform)**

| Query # | Query | Result |
| --- | --- | --- |
| 1. | (Vaccinium macrocarpon OR cranberry OR cranberry extract OR cranberry juice).mp | 3117 |
| 2. | (Urinary Tract Infections OR Tract Infections, Urinary OR Infection, Urinary Tract OR Tract Infection, Urinary OR Urinary Tract Infection OR Infections, Urinary Tract).mp | 139,903 |
| 3. | 1 AND 2 | 934 |

**Cochrane Database of Systematic Reviews (Ovid platform)**

| Query # | Query | Result |
| --- | --- | --- |
| 1. | MeSH descriptor: [[Vaccinium macrocarpon](" \l "0" \o "Phrase Matches)] explode all trees | 151 |
| 2. | (Cranberry or Cranberries or macrocarpon, Vaccinium or Vaccinium macrocarpons):ti,ab,kw | 479 |
| 3. | 1 OR 2 | 479 |
| 4. | MeSH descriptor: [Urinary Tract Infections] explode all trees:ti,ab,kw | 10164 |
| 5. | (Tract Infections, Urinary or Infection, Urinary Tract or Tract Infection, Urinary or Urinary Tract Infection or Infections, Urinary Tract):ti,ab,kw | 10165 |
| 6. | 4 AND 5 | 10165 |
| 7. | 3 AND 6 | 218 |
